# Colostrum avoidance practice and associated factors among mothers of children aged less than six months in Bure District, Amhara Region, North West, Ethiopia: A community-based cross-sectional study

**DOI:** 10.1371/journal.pone.0245233

**Published:** 2021-01-29

**Authors:** Ayenew Mose, Merga Dheresa, Bezatu Mengistie, Biresaw Wassihun, Haimanot Abebe

**Affiliations:** 1 Department of Midwifery, College of Medicine and Health Sciences, Wolkite University, Wolkite, Ethiopia; 2 School of Nursing and Midwifery, Colleges of Health Sciences and Medicine, Haramaya University, Harar, Ethiopia; 3 School of Public Health, Colleges of Health Sciences and Medicine, Haramaya University, Harar, Ethiopia; 4 Department of Midwifery, College of Medicine and Health Sciences, Arba Minch University, Arba Minch, Ethiopia; 5 Department of Public Health, College of Medicine and Health Sciences, Wolkite University, Wolkite, Ethiopia; Izmir Katip Celebi Universitesi Tip Fakultesi, TURKEY

## Abstract

**Introduction:**

The World Health Organization and the United Nations International Children’s Emergency Fund jointly recommend colostrum feeding immediately following delivery of the newborn. Colostrum avoidance is the practice of discarding colostrum at least once within the first three days after delivery of the newborn. Colostrum contains antibodies that protect the newborn against disease. Despite this fact, many Ethiopian mothers see colostrum feeding as a cause of neonatal morbidity and mortality, and they believe colostrum should be discarded to alleviate its effects. However, the cause of this misconception about colostrum is not well researched in Ethiopia, particularly in the study area.

**Objective:**

To assess the prevalence of colostrum avoidance and associated factors among mothers of children aged less than six months in Bure district, Amhara Region, North West, Ethiopia.

**Methods:**

A community-based cross-sectional study was conducted from March 1^st^ to 30^th^, 2019 in Bure district. Structured questionnaires and face to face interviews were used to collect data from 621 study participants. Multistage sampling technique was used to select study participants. Data were entered into Epi Data version 4.2.0 and then exported to Statistical Package for the Social Sciences version 23. Bivariate and multivariate logistic regression analyses were performed to identify predictors of colostrum avoidance practice.

**Results:**

Six hundred twenty-one (621) mothers of children aged less than six months participated. The prevalence of colostrum avoidance was 14.5% (95%CI; 11.5–17.4). The multivariate analysis indicated that home delivery [AOR = 3.350, (95%CI; 2.517–14.847)], giving birth through cesarean section [AOR = 3.368, (95%CI; 1.339–8.471)], no participation in an antenatal care group [AOR = 1.889, (95%CI; 1.144–3.533)], poor knowledge of mothers about colostrum [AOR = 3.44, (95%CI; 2.45–4.977)], and poor attitude of mothers towards colostrum [AOR = 3.053, (95%CI; 1.157–8.054)] were important predictors of colostrum avoidance practice.

**Conclusion and recommendations:**

Home delivery, giving birth through cesarean section, no participation in an antenatal care group, poor knowledge of mothers about colostrum, and poor attitude of mothers towards colostrum were significantly associated with colostrum avoidance practice. Therefore, health care workers in the district should promote institutional delivery, reduce the magnitude of cesarean section, encourage pregnant mothers to participate in an antenatal care group, and enhance maternal awareness about the merits of colostrum feeding. Moreover, health educations for mothers to have a positive attitude towards colostrum are important recommendations to be taken to prevent the further practice of colostrum avoidance.

## Introduction

The World Health Organization (WHO) and the United Nations International Children’s Emergency Fund (UNICEF) jointly recommend colostrum feeding immediately following delivery of the newborn. Colostrum avoidance is to discard colostrum at least once in the first three days of early neonatal life. According to WHO, Colostrum is defined as the first milk, thick, sticky, and clear to yellowish colour secreted during the first hour after birth up to three days. It is also called ‘***liquid gold’*** and ***‘passport of life’*** due to its highest content of antibodies as compared to mature breast milk [1].

Colostrum is the perfect first food for the newborn. It has significant advantages for newborns and mothers. For the newborns; firstly, it contains antibodies such as IgA, IgG, and IgM that protect against bacterial, viral, and fungal infections [2, 3]. Secondly, it is vital for the optimal growth and development of the newborns and further reduces the risk of early neonatal malnutrition. Thirdly, it contains growth factors such as platelet-derived growth factor (PDGF) and epidermal growth factors (EGF) that are used to mature the newborn’s intestine and that facilitates the passage of meconium, and further it reduces excess bilirubin and prevents neonatal jaundice. For the mothers, early colostrum feeding stimulates oxytocin release and causes the uterus to contract and reduce postpartum hemorrhage after birth [4–6].

Ethiopian national strategy on infant and young children feeding guidelines in 2004 considered colostrum feeding as the first neonatal immunization. Despite this, colostrum avoidance is still practiced; for instance, nationally more than 40% of mothers avoid colostrum [5, 7]. Colostrum avoidance varied across different districts, which showed that a high level of colostrum avoidance in Kossoye, Northern, rural part of Amhara 79% [8] and Afambo district 35% [9]. But, low levels of colostrum avoidance were reported in Axum town 6.4% [10] and Hula district 4% [11]. Based on the above findings, low levels of colostrum avoidance for those mothers who had antenatal care visits, postnatal visits, and gave birth in the hospital, and high levels of colostrum avoidance for those mothers who did not have antenatal care visits and lacked maternal education were reported [8–11].

Ethiopian mothers considered colostrum **‘‘*Inger”*** and breast milk **‘‘*Yetutwotet*”** as different substances. Many mothers think colostrum feeding is a cause of neonatal morbidity and mortality, and they believe colostrum should be discarded to alleviate its effects [8]. The cause of this misconception about colostrum is not well researched in Ethiopia, particularly in the study area. Ethiopian Demographic Health Survey in 2016 (EDHS) showed that 8% of infants were given prelacteal feedings within the first three days after birth [12]. However, it lacks assessment of prevalence and identifying factors related to colostrum avoidance practice. Similarly, there are few studies conducted in Ethiopia and there is no study specifically in the study area. Therefore, the main aim of this study was to assess the prevalence of colostrum avoidance and associated factors among mothers of children aged less than six months in Bure district. This study will have huge benefits for the district health office, health care workers, policymakers, and different stakeholders to eliminate further practicing colostrum avoidance and to upgrade optimal breastfeeding practice.

## Methods and materials

### Study design, period, and area

A community-based cross-sectional study design was employed in Bure district from March 1^st^ to 30^th^, 2019. Bure district is one of the 15 districts of West Gojjam Administrative Zone, Amhara Regional State, Ethiopia. It is found 148 km South West of Bahir Dar, capital of Amhara Regional State, and 400 km North West of Addis Ababa, the capital city of Ethiopia. The total population of the district was 132,614 of which 49.5% (65,644) are male and the remaining 50.5% (66,970) are female. There are five (5) functional health centres, four (4) medium private clinics, and twenty (20) health posts which are designed to provide primary health care services to the catchment population during the time of this study.

### Source and study population

The source populations were all mothers who had children less than six months of age in Bure district. Mothers who had children less than six months of age, permanent residents, and lived in the study area for at least six months were included in the study. Mothers who were critically ill and unable to respond during the data collection period were excluded.

### Sample size determination and sampling procedure

A single proportion formula was used to estimate the sample size required for the study. The sample size calculation assumed the proportion (p) estimated level of colostrum avoidance 14% [13]. By adding a non-response rate of 10%, considering the assumption of 95% confidence level, 4% margin of error, and design effect 2, the final sample size was 635 mothers. A multistage sampling technique was used to select the study participants in the community. Firstly; in the primary stage, Bure district has 24 kebele (22 rural and 2 urban kebele). From 24 kebele, 7 rural and 1 urban kebele were selected using a simple random sampling technique. Secondly; in the secondary stage, a census was conducted to identify those mothers having children less than six months of age in every eight kebeles using the help of community health extension workers. A total of 1269 mothers having children less than six months of age were found in the selected kebeles. A Sampling frame was prepared. Proportional allocation and systematic random sampling techniques were used to select 621 study participants.

### Data collection tool, procedure, and quality control

The questionnaires were adopted after reviewing the relevant literature [14, 15]. Data were collected by face to face interviews using a structured questionnaire. To ensure quality initially, the questionnaires were pretested 5% (31) and then Cronbach's Alpha was calculated by using SPSS window version 23.0 to test internal consistency (reliability) of the items (0.8). Data were collected using six nurses as data collectors and four BSc nurses as supervisors. Two-day training was given for both supervisors and data collectors.

### Study variables and measurements

In this study, the outcome variable was colostrum avoidance and the independent variables were socio-demographic characteristics, maternal health service utilization, knowledge of mothers about colostrum, and attitude of mothers towards colostrum. Colostrum avoidance practice is defined as those mothers who avoid or discard colostrum at least once within the first three days after delivery of the newborn [1].

Good knowledge of mothers about colostrum was defined by responses greater than or equal to 60% of knowledge related questions, and those mothers who responded to less than 60% coded as poor knowledge [14]. Similarly, if the mothers responded greater than or equal to 60% of attitude related questions, it was coded as a good attitude, and if the mothers responded to less than 60% coded as a poor attitude towards colostrum [14].

Prelacteal feeding is defined as mothers who gave fluid or food to the newborns except drugs, vitamins, minerals, and vaccines before breastfeeding initiation, usually in the first few days of life after delivery [12].

Antenatal care (ANC) is care provided to pregnant mothers to have a healthy mother and a healthy newborn at the end of pregnancy. It routinely has four visits; firstly, between 8 and 12 weeks; secondly, between 24 and 26 weeks; thirdly, at 32 weeks, and lastly between 36 and 38 weeks. The contents of health education during each visit are health promotion and disease prevention, care provision, birth preparedness, and complication readiness [16].

### Data processing and analysis

Data were entered into Epi Data version 4.2.0 and exported to SPSS version 23 software package for analysis. Descriptive analysis results were presented in the form of tables and texts using frequencies and summary statistics such as standard deviation and percentage. Bivariate logistic regression analysis was used to determine the association of each independent variable with the outcome variable. Chi-square test was performed to determine the association between the knowledge of mothers about colostrum and attitude of mothers towards colostrum. P values <0.25 [17] in bivariate logistic regression were included in multivariate logistic regression in order to control possible confounders. Adjusted odds ratio along with 95% CI was estimated to identify predictors for colostrum avoidance practice by using multivariate analysis in the binary logistic regression. In this study P-value < 0.05 was considered to declare a result as a statistically significant association.

### Ethics approval and consent to participate

Ethical clearance was obtained from Haramaya University College of Health and Medical Sciences Institutional Health Research Ethical Review Committee. An official letter was written to Bure district. Next, a cooperation letter was written to all kebeles on which the study was carried out. The study, purpose, procedure, and duration, possible risks, and benefits of participating in this study were clearly explained for each study participant using the local language (Amharic). Verbal and informed consent was taken from each study participant before data collection. The study participants have the right to withdraw from the study at any time during data collection.

## Results

### Socio-demographic characteristics

In this study, a total of 621 study participants were involved, making a response rate of 97.8%. The mean (±) age of study participants was 29.96 (± 6.149 SD) years. Of the total respondents, 304 (49%) aged from 22 to 29 years old and the majority of the respondents, 549 (88.4%) were married, 194 (31.2%) were could read and write without formal education, and 586 (94.3%) of the respondents were orthodox Christian religion followers. Out of the total respondents, 327 (52.7%) of mothers were housewives. Regarding the infant sex, half 314 (50.6%) of the infants were female (Table 1).

**Table 1:** Socio-demographic characteristics of mothers children aged less than six months in Bure District, Amhara Region, North West, Ethiopia, 2019 (n = 621).

Variable	Frequency	Percentage (%)
**Maternal age**		
≤21	40	6.4
22–29	304	49.0
30–38	192	30.9
39+	85	13.7
**Maternal educational status**		
Unable to read and write	140	22.5
Can read & write without formal education	194	31.2
Grade 1–8	130	20.9
Grade 9–12	116	18.7
College/University and above	41	6.6
**Maternal household head**		
Yes	160	25.8
No	461	74.2
**Fathers occupation**		
Unemployed	91	14.7
Employed	530	85.3
**Infant sex**		
Male	307	49.4
Female	314	50.6
**Maternal religion**		
Orthodox	586	94.4
Protestant	28	4.5
Muslim	7	1.1
**Ethnicity**		
Amhara	589	94.8
Oromo	32	4.2

### Maternal health care service utilization

In this study, 366 (58.9%) of mothers were multiparous. The majority of mothers had ANC follow up 456 (73.4%), 186 (30%) of them utilized ANC four times, and 349 (56.2%), of them had breastfeeding counselling at ANC clinic. The majority of mothers gave birth through vaginal delivery 565 (90.5%). Among 621 study participants, 11.6% (95% CI: 9.0–14.2) of mothers gave prelacteal feeding to the newborn during the first few days after delivery. Of these, 23 (3.7%) of mothers gave equally fresh butter and plain water for their newborns following delivery. The main reason for practicing prelacteal feeding was delayed initiation of breastfeeding 38 (6.1%) (Table 2).

**Table 2:** Maternal health care service utilization among mothers of children aged less than six months in Bure District, Amhara Region, North West, Ethiopia, 2019 (n = 621).

Variable	Frequency	Percentage (%)
**Parity**		
Primiparous	255	41.1
Multiparous	366	58.9
**Antenatal care visit**		
Yes	456	73.4
No	165	26.6
**Number of antenatal care visit**		
1	84	13.5
2	103	16.6
3	83	13.4
≥4	186	30
**Breastfeeding counselling during ANC visit**		
Yes	349	56.2
No	107	17.2
**Place of delivery**		
Health institution	564	90.8
Home	57	9.2
**Mode of delivery**		
Vaginal	568	91.5
Cesarean section	53	8.5
**Breastfeeding initiation time**		
Within one hour	507	81.6
After one hour	114	18.4
**Prelacteal feeding**		
Yes	72	11.6
No	549	88.4
**Type of prelacatel feeding**		
Plain water	23	3.7
Fresh butter	23	3.7
Cow milk	14	2.3
Honey	7	1.1
Tea	5	0.8
**Reason of prelacteal feeding**		
Delayed initiation of breastfeeding	38	6.1
Cultural practice	18	2.9
Inadequate breast milk secretion	9	1.5
Maternal illness	7	1.1
**Neonatal illness**		
Yes	151	24.3
No	470	75.7
**Participation in an antenatal care group**		
Yes	278	44.8
No	343	55.2
**Postnatal care visit**		
Yes	501	80.7
No	120	19.3

### Knowledge and attitude of respondents towards colostrum

Four hundred fifty- nine (73.9%) of mothers had good knowledge and 162 (26.1%) of mothers had poor knowledge about colostrum. The majority 526 (84.7%) of mothers had good attitude and 95 (15.3%) of mothers had poor attitude towards colostrum (Table 3). The correlation between the knowledge of mothers about colostrum and attitude of mothers towards colostrum was significantly and positively associated at Pearson correlation coefficient (r = 0.389; P <0.01).

**Table 3:** Bivariate and multivariate logistic regression analysis of colostrum avoidance and its explanatory variables in Bure District, Amhara Region, North West, Ethiopia,2019(n = 621).

Variables	Colostrum avoidance		COR (95% CI)	AOR (95% CI)
	No	Yes		
**Maternal educational status**				
Unable to read and write	110(17.7%)	30(4.8%)	1.00	1.00
Can read & write without formal education	170(27.4%)	24(3.9%)	0.518(0.288–0.932)*	2.678(0.820–8.747)
Grade 1–8	110(17.7%)	20(3.2%)	0.667(0.357–1.245)	2.378(0.705–8.021)
Grade 9–12	103(16.6%)	13(2.1%)	0.463(0.229–0.936)*	1.615(0.402–6.479)
College/University and above	38(6.1%)	3(0.5%)	0.289(0.084–1.003)	2.641(0.494–14.112)
**Parity**				
Parous	213(34.3%)	42(6.8%)	1.306(0.834–2.047)	0.494(0.233–1.051)
Multiparous	318(51.2%)	48(7.7%)	1.00	
**Fathers occupation**				
Unemployed	65(10.4%)	26(4.2%)	2.912(1.724–4.921)*	0.942(0.309–2.870)
Employed	466(75%)	64(10.3%)	1.00	1.00
**Prelacteal feeding**				
Yes	45(7.2%)	27(4.3%)	4.6 (2.668–7.930)*	2.522(0.965–4.966)
No	486(78.2%)	63(10%)	1.00	1.00
**Breastfeeding initiation**				
Within one hour	459(73.9%)	48(7.7%)	1.00	1.00
After one hour	72(11.6%)	42(6.8%)	5.578(3.442–9.041)*	1.967(0.948–4.428)
**Breastfeeding counselling during antenatal care visit**				
Yes	326(52.5%)	23(3.7%)	1.00	1.00
No	88(14.2%)	19(3.05%)	3.060(1.595–5.872)*	2.944(0.824–3.488)
**Place of delivery**			.	
Health institution	498(80.2%)	66(10.6%)	1.00	1.00
Home	33(5.3%)	24(3.9%)	5.488(3.057–9.852)**	3.350(1.248–15.167)*
**Mode of delivery**				
Vaginal delivery	494(79.5%)	74(11.9%)	1.00	1.00
Cesarean section	37(5.95%)	16(2.6%)	2.887(1.529–5.449)*	3.368(1.339–8.471)*
**Neonatal illness**				
Yes	123(19.8%)	28(4.5%)	1.00	1.00
No	408(65.7%)	62(9.9%)	0.668(0.409–1.089)	0.549(0.226–1.33)
**Participation in an antenatal care group**				
Yes	261(42%)	17(2.7%)	1.00	1.00
No	270(43.5%)	73(11.8%)	4.151(2.384–7.228)*	1.889(1.144–3.533)*
**Postnatal care visit**				
Yes	445(71.7%)	56(9%)	1.00	1.00
No	86(13.8%)	34(5.5%)	3.14(1.935–5.101)*	0.576(0.180–1.846)
**Knowledge**				
Good knowledge	419(67.5%)	40(6.4%)	1.00	1.00
Poor knowledge	112(18%)	50(8.1%)	4.676(2.937–7.446)*	3.44(2.45–4.977)*
**Attitude**				
Good attitude	480(77.3%)	46(7.4%)	1.00	1.00
Poor attitude	51(8.2%)	44(7.1%)	9.003(5.438–14.904)**	3.053(1.157–8.054)**

Significant at **P -Value <0.*001*, *P-Value <0.*05*, *1*.*00 = reference*, *Hosmer-Leme show goodness-of-fit = 0*.*798*.

### Colostrum avoidance practice

The prevalence of colostrum avoidance practice among mothers having children less than six months of age within three days after delivery was 14.5% (95% CI: 11.8–17.3). The major reason given by respondents for colostrum avoidance was due to colostrum being a dirty part of breast milk 26 (4.2%). Giving colostrum causes neonatal illness 14 (2.3%), colostrum is culturally forbidden 24 (3.9%), colostrum is not good for neonatal health 9(1.4%), and colostrum is very thick 17 (2.7%) were mentioned by the remaining mothers who had practiced colostrum avoidance.

### Factors associated with colostrum avoidance

The multivariate analysis indicated that home delivery, giving birth through cesaeran section, no participation in an antenatal care group, poor knowledge of mothers about colostrum and poor attitude of mothers towards colostrum were significantly associated with colostrum avoidance practice.

The odds of practicing colostrum avoidance were 3.35 times higher among mothers who had given birth at home compared to those who had given birth at a health institution (AOR = 3.35, 95%CI: 1.248–15.167). The odds of practicing colostrum avoidance were 3.368 higher among mothers who had given birth through cesarean section compared to those who had given a vaginal delivery (AOR = 3.368, 95%CI: 1.339–8.471). The odds of practicing colostrum avoidance were 1.889 times higher among mothers who had not participated in an antenatal care group compared to those who did participate in an antenatal care group (AOR = 1.889, 95% CI: 1.144–3.533). Those mothers who had poor knowledge of colostrum were 3.44 times more likely to practice colostrum avoidance compared to those mothers who had good knowledge of colostrum (AOR = 3.44, 95% CI: 2.45–4.977). The odds of practicing colostrum avoidance were 3.053 times higher among mothers who had a poor attitude towards colostrum compared to those mothers who had a good attitude towards colostrum (AOR = 3.053, 95%CI: 1.157–8.054) (Table 3).

## Discussion

It is well known that early initiation of breastfeeding immediately following delivery of the neonate has a huge benefit for neonatal and maternal health. Despite this fact, there is a significant proportion of mothers who practiced colostrum avoidance in the study area. This study found that the prevalence of colostrum avoidance practice among mothers having children less than six months of age was 14.5% (95% CI; 11.5–17.4). Home delivery, giving birth through cesaeran section, no participation in an antenatal care group, poor knowledge, and poor attitude of mothers towards colostrum were significantly associated with colostrum avoidance practice.

The prevalence of colostrum avoidance in this study is in line with studies done in Shire Endaslassie town 14% [14] and Raya Kobo district 13.5% [17]. It is higher than studies done in Aksum town 6.4% [10], Kombolcha town 11.4% [18], and North Wollo Zone 12% [19]. However, it is lower than studies conducted in Boset district, East-central Ethiopia 42% [15], and central India 23% [20]. The possible explanation for the difference might be due to socio-cultural variations of the infant breastfeeding practice, maternal health service access, and geographical distribution of study participants.

Home delivery was found to be significantly associated with colostrum avoidance practice. Mothers who gave birth at home were 3.35 times more likely to practice colostrum avoidance compared to those mothers who gave birth at a health institution, which is consistent with studies conducted in Aksum town [10], Raya Kobo district [17], and North Wollo Zone [19]. The possible reason could be that those mothers who gave birth at home are influenced by their family members, relatives, and Traditional Birth Attendants (TBAs). In this study, mothers who gave birth through cesarean section were 3.368 times more likely to avoid colostrum than those who had a vaginal delivery. The possible reason for this could be due to mothers who gave birth through cesarean section would be at a high risk of postpartum infections and the side effect of anaesthesia that might separate mothers from infants. Therefore, during this separation mothers might discard colostrum. Mothers who did not participated in an antenatal care group were 1.889 times more likely to practice colostrum avoidance as compared to mothers who participate in an antenatal care group, which is congruent with a study done in Kombolcha town [18]. The reason might be during participation in an antenatal care group with health care workers, mothers increase their awareness about the optimal breastfeeding practice such as the advantage of early initiation of breastfeeding within one hour after birth.

Mothers who had poor knowledge of colostrum were 3.44 times more likely to practice colostrum avoidance compared to mothers who had good knowledge of colostrum. This was congruent with recent studies conducted in Aksum town [10] and Raya kobo district [17]. This might be a result of mothers who lack knowledge about the importance of colostrum feeding to their newborns choose to avoid colostrum. On the other hand, mothers who had poor attitudes towards colostrum were 3.053 times more likely to practice colostrum avoidance compared to those mothers having a good attitude towards colostrum. The possible explanation for this might be mothers having poor attitude towards colostrum have considered colostrum feeding as a cause of neonatal infections and even neonatal morbidity and mortality. In the present study, colostrum is avoided primarily because mothers consider it as a dirty part of breast milk which is consistent with a study conducted in Kombolcha town [18].

The Pearson correlation coefficient results show that there is a positive and significant association between the knowledge of mothers about colostrum and the attitude of mothers towards colostrum with colostrum avoidance practice. Therefore, it could be established that those mothers who had more knowledge about colostrum are more likely motivated to have a positive attitude towards colostrum feeding to their newborns.

## Conclusion and recommendation

Home delivery, giving birth through cesarean section, no participation in an antenatal care group, poor knowledge of mothers about colostrum, and poor attitude of mothers towards colostrum were independent predictors of colostrum avoidance practice. Therefore, health care workers should promote institutional delivery, reduce the magnitude of cesarean section, encourage pregnant mothers to participate in an antenatal care group, and enhance maternal knowledge about the merits of colostrum feeding. Moreover, health educations for mothers to have a positive attitude towards colostrum are important recommendations to be taken to prevent the further practice of colostrum avoidance.

## Limitation of the study

This study shares some limitations. One of the limitations was recall bias due to study participants unable to remember what happened in the past six months. The second one is the lack of qualitative data supplementations and it also shares limitations of cross-sectional study design.

## Supporting information

S1 File English version questionnaire. (DOCX)

S2 File Amharic version questionnaire. (DOCX)

S3 File Minimal data set. (SAV)

## Abbreviations

ANC: Antenatal Care
AOR: Adjusted Odd Ratio
COR: Crude Odd Ratio
SPSS: Statistical Package for Social Sciences
UNCIEF: United Nations International Children’s Emergency Fund
WHO: World Health Organization
