# Supplementary material for: Colostrum avoidance practice and associated factors among mothers of children aged less than six months in Bure District, Amhara Region, North West, Ethiopia: A community-based cross-sectional study
Source: PLoS One. 2021 Jan 29;16(1):e0245233. doi: 10.1371/journal.pone.0245233 (PMC7846012; doi:10.1371/journal.pone.0245233)
Supplement: S1 File — (DOCX) [file pone.0245233.s001.docx]

**English Version Questionnaire**

Name of interviewer: _______________Signature ____________ Date: ___________

Date of interview_____|_____| ______| Code ----------

Questionnaire adopt to assess colostrum aviodance and associated factor among mothers of childeren age less than six months in Bure District ,North West, Amhara Region, Ehiopia 2019.

| **Part I: Socio- demographic characteristics** | | | | | | | | | | | | | | | |
| --- | --- | --- | --- | --- | --- | --- | --- | --- | --- | --- | --- | --- | --- | --- | --- |
| S. No. | | | | Item | | | | Response | | | | Skip | | | |
| 101 | | | | How old are you? | | | | ………./in complete year/ | | | |  | | | |
| 102 | | | | What is your current marital status? | | | | 1.Married  2.Single  3.Divorced  4.Widowed | | | |  | | | |
| 103 | | | | What is your religion? | | | | 1.Orthodox  2. Protestant  3. Catholic  4. Muslim | | | |  | | | |
| 104 | | | | What is your educational status? | | | | 1.Unable to read and write  2.Can read & write without formal education  3.Grade 1-8  4.Grade 9-12  5.College/University and above | | | |  | | | |
| 105 | | | | Mother household head | | | | 1.Yes  2.No | | | |  | | | |
| 106 | | | | What is your ethnicity? | | | | 1.Amhara  2.Oromo | | | |  | | | |
| 107 | | | | Father’s occupation? | | | | 1.Employed  2.Unemployed | | | |  | | | |
| 108 | | | | Infant sex | | | | 1. Male | | | |  | | | |
|  |  |  |  |  |  |  |  | 1. Female | | | |  | | | |
| **Part II: Colostrum avoidance practice** | | | | | | | | | | | | | | | |
| 201 | | Did you avoid colostrum during breastfeeding 1 up to 3 day after birth? | | | 1.Yes | | | | | | | If ‘No’ skip to Q203 | | | |
|  |  |  |  |  | 2.No | | | | | | |  |  |  |  |
| 202 | | If yes for the above question, why you avoid colostrum? | | | 1. It cause illness for neonates 2. It is Culturally forbidden 3. It is dirty part of breast milk 4. It is not good for neonates health 5. It is very thickness | | | | | | |  | | | |
| 203 | | Did you give pre-lacteal feeding? | | | 1. Yes 2. No | | | | | | | If ‘No’ skip to  Q 206 | | | |
| 204 | | If yes What type of feeding is given to the baby soon  After birth? | | | 1. Water 2. Butter 3. Honey 4. Cow milk 5. Tea | | | | | | |  | | | |
| 205 | | Why you give pre-lacteal feeding? | | | 1. Inadequate breast milk secretion 2. Delayed lactation 3. Cultural practice 4. Maternal illness 5. Infant feeding problem | | | | | | |  | | | |
| 206 | | When did you start Breast feeding initiation? | | | 1. Within 1 h | | | | | | |  | | | |
|  |  |  |  |  | 1. Greater than 1 h | | | | | | |  |  |  |  |
| **Part III:- Maternal Health Care Service Utilization** | | | | | | | | | | | | | | | |
| 301 | | Parity | | 1. Parous 2. Multiparous | | | | | | |  | | | |  |
| 302 | | Did you have ante natal care visit? | | 1. Yes 2. No | | | | | | | If ‘No’ skip to Q 305 | | | |  |
| 303 | | If yes for above question how many times you visit ANC? | | 1. 1 2. 2 3. 3 4. ≥ 4 | | | | | | |  | | | |  |
| 304 | | Did you receive counseling on breastfeeding during ANC Visit? | | 1. Yes 2. No | | | | | | |  |  |  |  |  |
| 305 | | Place of delivery? | | | 1. Health institution 2. Home | | | | | | |  | | | |
| 306 | | Mode of delivery? | | | 1. Vaginal 2. Cesarean section | | | | | | |  | | | |
| 307 | | Neonatal illness? | | | 1. Yes 2. No | | | | | | |  | | | |
| 308 | | Did you Participate antenatal care group? | | | 1. Yes 2. No | | | | | | |  |  |  |  |
| 309 | | Did you have postnatal care visit? | | | 1. Yes 2. No | | | | | | |  | | | |
| **Part IV: Knowledge related questions** | | | | | | | | | | | | | | | |
| 401 | | Did you ever heard about colostrum? | | | | 1. Yes 2. No | | | | | | | If ‘No’ skip to 501 questions | | |
| 402 | | From where did you get the information? | | | | 1. Health professionals 2. Family 3. Media 4. Friends | | | | | | |  | | |
| 403 | | Color of colostrums? | | | | 1. Yellow 2. Red 3. Others …………. | | | | | | |  | | |
| 404 | | Dose it protects infectious diseases? | | | | 1. Yes 2. No | | | | | | |  | | |
| 405 | | Colostrums important part of breast milk? | | | | 1. Yes 2. No | | | | | | |  | | |
| 406 | | Should child feed colostrums when you are sick? | | | | 1. Yes 2. No | | | | | | | If ‘yes’ skip to  Q 408 | | |
| 407 | | If no, above questions why? | | | | 1. it harms me 2. it harms baby 3. both it harms me and baby | | | | | | |  | | |
| 408 | | Did you feed colostrums when baby sick? | | | | 1. Yes 2. No | | | | | | |  | | |
| **Part V: Attitude related questions** | | | | | | | | | | | | | | | |
|  | |  | | | | | Strongly disagree | | Disagree | Nutral | Agree | | | Strongly  agree | |
| 501 | | Do you believe that the first milk (colostrum) should discard? | | | | | 1 | | 2 | 3 | 4 | | | 5 | |
| 502 | | Do you think that colostrum is not important for baby growth and development? | | | | | 1 | | 2 | 3 | 4 | | | 5 | |
| 503 | | Do you think that colostrum is difficult to digest and needs to be discarded | | | | | 1 | | 2 | 3 | 4 | | | | 5 |
| 504 | | Do you think colostrum is forbidden in culture? | | | | |  | |  |  |  | | | |  |
| 505 | | Do you think colostrum is dirty part of milk? | | | | | 1 | | 2 | 3 | 4 | | | | 5 |
| 506 | | Dose colostrum cause neonatal diarrhea? | | | | | 1 | | 2 | 3 | 4 | | | | 5 |
| 507 | | Do you think baby did not like colosrum breast milk | | | | | 1 | | 2 | 3 | 4 | | | | 5 |
| 508 | | Dose colostrum cause abdominal cramp? | | | | | 1 | | 2 | 3 | 4 | | | | 5 |

**Thank you!!**
