# Supplementary material for: Colostrum avoidance practice and associated factors among mothers of children aged less than six months in Bure District, Amhara Region, North West, Ethiopia: A community-based cross-sectional study
Source: PLoS One. 2021 Jan 29;16(1):e0245233. doi: 10.1371/journal.pone.0245233 (PMC7846012; doi:10.1371/journal.pone.0245233)
Supplement: S2 File — (DOCX) [file pone.0245233.s002.docx]

**ቃለ መጠይቅ**

መጠይቁ የተሰጠበት ቀን-----------------መጠይቁን የሞላው ሰው----------------------- የመጠይቁ መለያ ቁጥር -

| ተ/ቁ | | ጥያቄ | መልስ | | | | | | | | | | | | | | ይልፉ |
| --- | --- | --- | --- | --- | --- | --- | --- | --- | --- | --- | --- | --- | --- | --- | --- | --- | --- |
| **ክፍል አንድ : - ማህበራዊና የኋላ ታሪክ ባህሪ ላይ የሚያጠነጥኑ መጠይቆች** | | | | | | | | | | | | | | | | |  |
| 101 | ዕድሜዎ ምን ያህል ነው? | | | ዓመት | | | | | | | | | | | | |  |
| 102 | የጋብቻ ሁኔታ? | | | 1. ያገባ  2. ያላገባ  3. አግብቶ የፈታ  4. ባለቤቷ የሞተባትብ | | | | | | | | | | | | |  |
| 103 | ሃይማኖቶ ምንድን ነው? | | | 1. ኦርቶዶክስ  2. ፕሮቴስታንት  3. ሙስሉም | | | | | | | | | | | | |  |
| 104 | የትምህርት ደረጃ? | | | 1. ማንበብና መጻፍ የማትችል  2. ማንበብና መጻፍ የምትችል  3. የመጀመሪያ ደረጃ ትምህርት (1-8) ያጠናቀቀች  4.ሁለተኛ ደረጃ ትምህርት(9-12) ያጠናቀቀች  5. ኮሌጅና ከዛ በላይ | | | | | | | | | | | | |  |
| 105 | የቤቱ ሃላፈ እረሶዎ ኖዎት? | | | 1. አዎ 2. የለም | | | | | | | | | | | | |  |
| 106 | ከየት ክልል ኖዎት? | | | 1. አማራ  2. ኦሮሞ | | | | | | | | | | | | |  |
| 107 | ባለቤትዎ ስራ አለው? | | | 1. አለ  2. የለም | | | | | | | | | | | | |  |
| 108 | የልጁዎ ጾታ ምንድ ነው? | | | 1. ሴት  2. ወንድ | | | | | | | | | | | | |  |
| **ክፍል ሁለት : - እንግር አለማጥባት ላይ የሚያጠነጥኑ መጠይቆች** | | | | | | | | | | | | | | | | | |
| 201 | እንግር የመጅመረያው የእናት ጡት ወተት አላጠባሻም ? | | | | | | | 1. አዎ  2. አጥብቻለው | | | | | መልስ አጥብቻለው ክሆነ ወድ ጥያቄ 203 ይልፉ | | | | |
| 202 | ለ 201 ጥያቄ መልስ አዎ ክሆን ለምን አላጠባሽም? | | | | | | | 1.ህጻኑን ልህመም ስለሚያጋልጥ  2.በባህላችን ማጥባት ስለሚክለክል  3.እንግር ቆሻሻ ስለሆነ  4.እንግር ጥሩ አይድለም 5.ወፍራም ስለሆነ | | | | | | | | |  |
| 203 | ህጻኑ እድተወለድ ክእናት ጡት ወተት ዉጭ ስጠሽ ነበር? | | | | | | | 1. አዎ  2. የለም | | መልስ የለም ክሆነ ወድ ጥያቄ 206 ይልፉ | | | | | | | |
| 204 | ለ203 ጥያቄ መልስ አዎ ክሆን ምን አይነት ምግብ ነው የሰጠሽው/ሻት? | | | | | | | 1.ዉሃ  2.ቅቤ  3.ማር  4. የላም ወተት  5.ሻይ  6. ሌላ ካለ ይጠቀሱ……................. | | | | | | |  | | |
| 205 | ክ እናት ጡት ወተት ዉጭ የስጠሽበት ምክኒያት ምንድን ነው? | | | | | | | 1.ጡት ወተት ብቻ ስለማይበቃው  2.ጡት ወተት ቶሎ አልወጣ ስለአለ  3.ባህል ስለሆነ  4.አሞኝ ስለነበር  5.ህጻኑ በደንብ መጥባት ስለአልቻለ | | | | | | |  | | |
| 206 | ጡት ወተት ማጥባት እንድ ወለድሽ በስንት ስዓት ጅመረሽ? | | | | | | | 1. በ 1 ስዓት ውስጥ  2.ክ 1 ስዓት በኃላ | | | | | | | |  | |
| **ክፍል ሶስት፡- የወሊድ ሁኔታ እና ጤና ተቋም አጠቃቅም ላይ የሚያጠነጥኑ መጠይቆች** | | | | | | | | | | | | | | | | | |
| 301 | ይሄ ህጻን ስንተኛ ልጅዏ ነው? | | | | | | 1 .የመጅመረያ  2. ሁለተኛና ክዛ በላይ | | | |  | | | | | | |
| 302 | የእናቶች የነፍሰ ጡር ክትትል አረገሽ ነበር? | | | | | | 1. አዎ  2. የለም | | | | መልስ **የለም** ክሆነ ወድ ጥያቄ **305** ይልፉ | | | | | | |
| 303 | ለ **302** ጥያቄ መልስ **አዎ** ክሆን ስንት ጊዜ ተክታተልሽ? | | | | | | 1. 1  2. 2  3. 3  4. >4 | | | |  | | | | | | |
| 304 | ስለጡት ማጥባት ምክር ተሰጦሽ ነበር? | | | | | | 1. አዎ  2. የለም | | | |  | | | | | | |
| 305 | የመጨረሻ ጊዜ ልጅዎን የወለዱት የት ነበር? | | | | | | 1.ጤና ተቋም  2. ቤት | | | |  | | | | | | |
| 306 | የመጨረሻ ልጅዎን የወለዱት እንዴት ነበር? | | | | | | 1. በመህፀን  2. በኦፕሬሽን | | | |  | | | | | | |
| 307 | የመጨረሻው ልጅዎ እደተወለደ ታሞ ነበር? | | | | | | 1. አዎ  2. የለም | | | |  | | | | | | |
| 308 | የነፍሰጡር እናቶች የምክክር ፕሮግራም ተሳትፈሽ ታውቂያለሽ? | | | | | | 1. አዎ  2. የለም | | | |  | | | | | | |
| 309 | የድህረ ወሊድ ክትትል አድርገዋል? | | | | | | 1. አዎ  2. የለም | | | |  | | | | | | |
| **ክፍል አራት፡- የእናትዮዋን ዕውቀት ለመዳሰስ የተዘጋጁ መጠይቆች** | | | | | | | | | | | | | | | | | |
| 401 | ስለ እንግር የእናት ጡት ወተት ክዚህ በፈት ስምተሽ ታውቂያለሽ? | | | | | 1. አዎ  2. የለም | | | | | | | | መልስ **የለም** ክሆነ ወድ ጥያቄ **501** ይልፉ | | | |
| 402 | ለ **401** ጥያቄ መልስ **አዎ** ክሆን ክየት ነው የሰማሽው? | | | | | 1. ጤና ባለሙያ  2. ቤተሰብ  3. ክመገናኛ ቡዙሀን  4. ክጎደኛ | | | | | | | |  | | | |
| 403 | እንግር ምን አይነት ቀለም አለው? | | | | | 1.ቢጫ  2. ቀይ  3 ሌላ ካለ ይግለጹ------ | | | | | | | |  | | | |
| 404 | እንግር ክበሽታ ልጁን ይክላክላል? | | | | | 1. አዎ  2. የለም | | | | | | | |  | | | |
| 405 | እንግር በጣም ጠቃሚ የእናት ጡት ወተት ነው? | | | | | 1. አዎ  2. የለም | | | | | | | |  | | | |
| 406 | አንቺን እንድውለድሽ አሞሽ ቢሆን እንግር ልጅ መጥባት አለበት? | | | | | 1. አዎ  2. የለም | | | | | | | | መልስ  **አዎ** ክሆነ ወድ ጥያቄ **408** ይልፉ | | | |
| 407 | ለ **406** መልስ **የለም** ክሆነ ለምን? | | | | | 1.እኔን ስለሚጐዱኝ  2.ልጅን ስለሚጐዳ  3.እኔንም ልጅንም ስለሚጐዳን | | | | | | | |  | | | |
| 408 | ልጅ ሲታመም “አንግር” መጥባት አለበት? | | | | | 1. አዎ  2. የለም | | | | | | | |  | | | |
| **ክፍል አምስት፡- የእናትዮዋን አመለካክት ለመዳሰስ የተዘጋጁ መጠይቆች** | | | | | | | | | | | | | | | | | |
|  |  | | | | በጣም  አልስማማም | | | | አልስማማም | | አልወስንም | እስማማለሁ | | | | | በጣም  እስማማለሁ |
| 501 | የመጅመረያ የእናት ጡት ወተት “እንግር” ልጁ መጥባት የለበትም ብለሽ ታስቢያለሽ? | | | | 1 | | | | 2 | | 3 | 4 | | | | | 5 |
| 502 | “እንግር” ለልጁ እድገት ጥቅም የለውም ብለሽ  ታስቢያለሽ? | | | | 1 | | | | 2 | | 3 | 4 | | | | | 5 |
| 503 | “እንግር”ለህጸኑ በድንብ ስለማይዋጥለት መጥባት የለበትም ብለሽ ታስቢያለሽ? | | | | 1 | | | | 2 | | 3 | 4 | | | | | 5 |
| 504 | “እንግር” ማጥባት በባህል የተክለከለ ነው ብለሽ ታስቢያለሽ? | | | | 1 | | | | 2 | | 3 | 4 | | | | | 5 |
| 505 | “እንግር” ከእናት ጡት ወተት ቆሻሻ ክፍሉ ነው ብለሽ ታስቢያለሽ? | | | | 1 | | | | 2 | | 3 | 4 | | | | | 5 |
| 506 | “እንግር” ልጁ ላይ ተቅማት ያመጣል ብለሽ ታምኛለሽ? | | | | 1 | | | | 2 | | 3 | 4 | | | | | 5 |
| 507 | ህጸኑ“አንግር”ን አይውድውም ብለሽ ታስቢያለሽ？ | | | | 1 | | | | 2 | | 3 | 4 | | | | | 5 |
| 508 | “እንግር” ልጁ ላይ የሆድ ህመም ያመጣል ብለሽ ታስቢያለሽ? | | | | 1 | | | | 2 | | 3 | 4 | | | | | 5 |

**አመሰግናለሁ**
